# An efficient molybdenum disulfide/cobalt diselenide hybrid catalyst for electrochemical hydrogen generation

**DOI:** 10.1038/ncomms6982

**Published:** 2015-01-14

**Authors:** Min-Rui Gao, Jin-Xia Liang, Ya-Rong Zheng, Yun-Fei Xu, Jun Jiang, Qiang Gao, Jun Li, Shu-Hong Yu

**Affiliations:** 1 Division of Nanomaterials & Chemistry, Hefei National Laboratory for Physical Sciences at Microscale, Collaborative Innovation Center of Suzhou Nano Science and Technology, Department of Chemistry, University of Science and Technology of China, Hefei 230026, China; 2 Key Laboratory of Organic Optoelectronics and Molecular Engineering of Ministry of Education, Department of Chemistry, Tsinghua University, Beijing 100084, China; 3 Guizhou Provincial Key Laboratory of Computational Nano-Material Science, Guizhou Normal College, Guiyang 550018, China

## Abstract

The electroreduction of water for sustainable hydrogen production is a critical component of several developing clean-energy technologies, such as water splitting and fuel cells. However, finding a cheap and efficient alternative catalyst to replace currently used platinum-based catalysts is still a prerequisite for the commercialization of these technologies. Here we report a robust and highly active catalyst for hydrogen evolution reaction that is constructed by *in situ* growth of molybdenum disulfide on the surface of cobalt diselenide. In acidic media, the molybdenum disulfide/cobalt diselenide catalyst exhibits fast hydrogen evolution kinetics with onset potential of −11 mV and Tafel slope of 36 mV per decade, which is the best among the non-noble metal hydrogen evolution catalysts and even approaches to the commercial platinum/carbon catalyst. The high hydrogen evolution activity of molybdenum disulfide/cobalt diselenide hybrid is likely due to the electrocatalytic synergistic effects between hydrogen evolution-active molybdenum disulfide and cobalt diselenide materials and the much increased catalytic sites.

## Abstract (web summary)

There is substantial research into new catalysts for electroreduction of water. Here, the authors report a robust and active molybdenum disulfide/cobalt diselenide hydrogen evolution catalyst with onset potential of 11 mV and Tafel slope of 36 mV per decade, approaching the activity of platinum.

## Graphical abstract

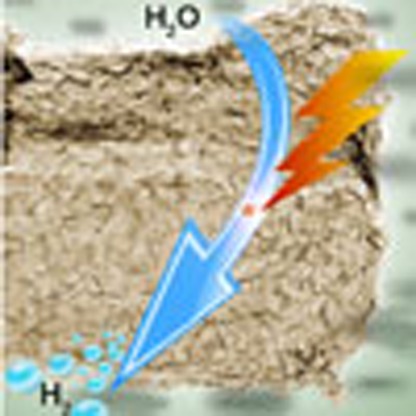

Owing to their diffuse nature, electricity from renewable but intermittent energy (for example, solar and wind) must be stored durably for off-grid applications. Electrochemical water splitting to produce hydrogen (H_2_) offers a promising and sustainable solution for this purpose by converting such electricity energy into stable chemical bonds^1^,^2^. Appropriate electrocatalysts, such as platinum (Pt) and its alloys, play a vital role in the H_2_ evolution reaction (HER) because they can catalyse the conversion from a pair of protons and electrons to H_2_ at high reaction rates and low overpotentials (*η*)^1^,^2^,^3^. However, the prohibitive cost and scarcity of Pt pose tremendous limitations to widespread use. Therefore, finding robust and efficient alternative catalysts that are geologically abundant is crucial to the future of ‘hydrogen economy’.

Molybdenum disulfide (MoS_2_), a widely used industrial catalyst for hydrodesulfurization^4^, has recently demonstrated promise as effective HER catalyst based on both computational and experimental studies^5^,^6^. The HER activity was discovered to arise from the exposed (10–10) planes on edges of MoS_2_, whereas the (0001) basal planes are catalytically inactive^5^,^6^,^7^. This understanding has led to great efforts to develop highly nanostructured MoS_2_-based HER catalysts to maximize the number of edge sites, including crystalline^8^,^9^,^10^,^11^,^12^ and amorphous materials^13^,^14^,^15^, MoS_2_-based hybrid materials^16^,^17^,^18^,^19^ and molecular mimics^7^. Despite significant success, the design and fabrication of MoS_2_-based HER electrocatalysts with satisfactory activity and stability remain a big challenge.

In recent years, we have been making efforts to explore efficient electrocatalysts by using Earth-abundant 3*d* metal (Co, Ni and so on) chalcogenides^20^,^21^,^22^,^23^,^24^,^25^,^26^,^27^. New NiSe nanofibres^24^ and lamellar mesostructured CoSe_2_/DETA (DETA, diethylenetriamine) nanobelts^25^ were found to show decent HER activity in acidic electrolyte. Kong *et al.*^28^ also observed good HER activities from various polycrystalline transition metal dichalcogenide (ME_2_, M=Fe, Co, Ni; E=S, Se) films, especially from the CoSe_2_. Recently, we found that the HER activity of CoSe_2_ nanobelts can be improved greatly after anchoring Ni/NiO nanoparticles onto their surfaces^25^. The synergetic chemical coupling effects between CoSe_2_ and grafted Ni/NiO were believed to contribute to the enhancement. Similar promoted performances have also been observed on MoS_2_/graphene^16^,^17^, MoO_3_/MoS_2_ (ref. 18) and MoS_2_/Au (ref. 19) composite catalysts for H_2_ production. These works point to the possibility to access new and efficient HER catalysts by combining the promising CoSe_2_ and MoS_2_.

We report here that a HER electrocatalyst based on quasi-amorphous MoS_2_-coated CoSe_2_ (denoted as MoS_2_/CoSe_2_) hybrid is highly active and stable in acidic electrolyte. Notably, without any noble metals, the MoS_2_/CoSe_2_ hybrid catalyst shows an onset potential close to commercial Pt catalyst (Johnson-Matthey, 20 wt% Pt/XC-72) and a small Tafel slope of ~36 mV per decade as well as no current loss after long-term chronoamperometry measurement, performing the best among the noble-metal-free HER electrocatalysts. These results suggest a strategy for designing non-noble metal catalysts with enhanced HER performance that is comparable to the state-of-the-art Pt-based catalysts.

## Results

### MoS_2_/CoSe_2_ hybrid catalyst

**Figure 1:**
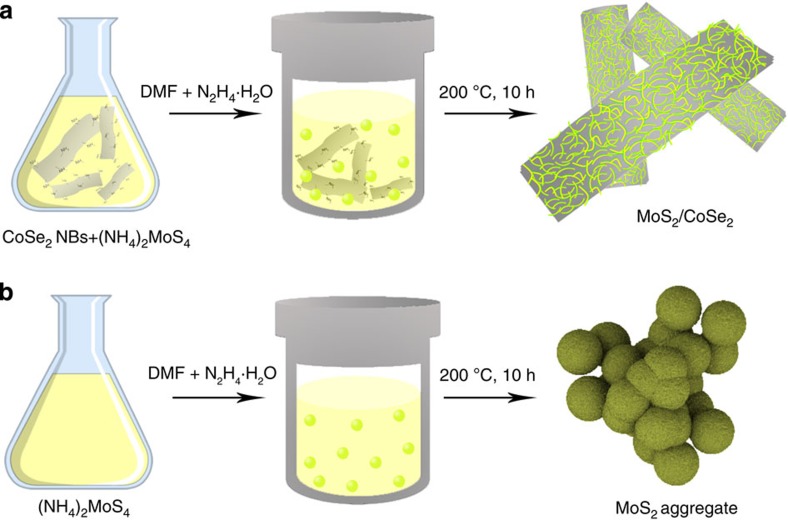
Schematic illustration of the preparation of MoS_2_/CoSe_2_ hybrid. (**a**) Solvothermal synthesis with CoSe_2_/DETA nanobelts as substrates for preparation of MoS_2_/CoSe_2_ hybrid. (**b**) Solvothermal synthesis without CoSe_2_/DETA nanobelts leads to free MoS_2_ nanosheet aggregates.

**Figure 2:**
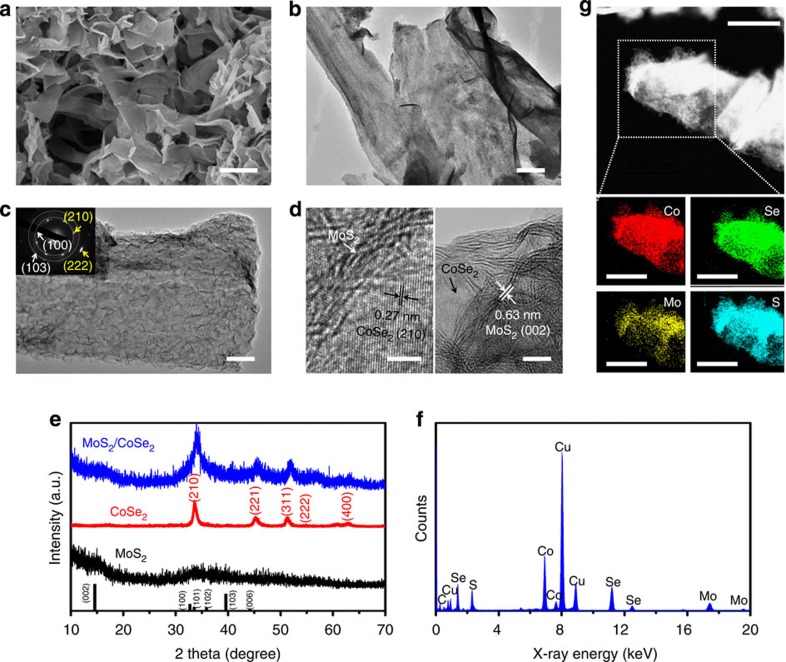
Characterization of the MoS_2_/CoSe_2_ hybrid. (**a**) Scanning electron microscopy image of MoS_2_/CoSe_2_ hybrid. Scale bar, 800 nm. (**b**,**c**) TEM images with different magnifications of MoS_2_/CoSe_2_ hybrid. Scale bars, 200 and 50 nm, respectively. The inset in **c** shows corresponding SAED pattern. (**d**) HRTEM images of MoS_2_/CoSe_2_ hybrid showing distinguishable microstructures of MoS_2_ and CoSe_2_. Scale bars, 5 nm. (**e**,**f**) XRD patterns and EDX spectrum of the MoS_2_/CoSe_2_ hybrid, respectively. (**g**) STEM-EDX elemental mapping of MoS_2_/CoSe_2_ hybrid showing clearly the homogeneous distribution of Co (red), Se (green), Mo (yellow) and S (azure). Scale bars, 200 nm.

The MoS_2_/CoSe_2_ hybrid was prepared directly in a closed *N*,*N*-dimethylformamide (DMF)/hydrazine solvothermal system, where (NH_4_)_2_MoS_4_ was used as a precursor for growing MoS_2_ around the freshly made CoSe_2_/DETA nanobelt substrates (Fig. 1a; Supplementary Fig. 1; see Methods for details of the synthesis). The MoS_2_-coated CoSe_2_ hybrid was shown by means of scanning electron microscopy and transmission electron microscopy (TEM; Fig. 2a–c), which revealed the compact graphene-like MoS_2_ nanosheets grown on the surface of CoSe_2_ with a partially free-standing branch-like feature (Supplementary Fig. 2). Substantial amino groups on the CoSe_2_/DETA surface (Supplementary Fig. 3) serve as nucleation sites for coupling Mo precursor and subsequently reduced to MoS_2_ on CoSe_2_ (refs 21, 23). A control experiment performed under identical synthesis conditions, but without CoSe_2_ nanobelts, produced three-dimensional (3D) aggregates of MoS_2_ sheets (Fig. 1b; Supplementary Fig. 4), suggesting that CoSe_2_ could be a useful support for mediating the growth of loaded materials and constructing novel functional hybrids.

Figure 2d presents high-resolution TEM (HRTEM) images of the MoS_2_/CoSe_2_ hybrid. Layered MoS_2_ (often less than five layers) nanosheets, with an interlayer separation of 0.63 nm, were grown intimately on the CoSe_2_ substrate. High-crystalline CoSe_2_ substrate with *d* spacing of 0.27 nm can be seen frequently through the interspace of grafted MoS_2_ (see Supplementary Fig. 5 for additional images). This non-fully covered structure may take advantage of merits from both MoS_2_ and CoSe_2_ for catalysing H_2_ evolution. Selected area electron diffraction (SAED) pattern (inset in Fig. 2c; Supplementary Fig. 6) revealed clear diffraction spots (marked by yellow arrows) from single-crystalline CoSe_2_ support (JCPDS 9-0234) and also faint diffraction rings from grafted MoS_2_ (JCPDS 77-1716). These barely recognizable diffraction rings indicate the quasi-amorphous structure of MoS_2_, consistent with the X-ray diffraction (XRD) data of MoS_2_/CoSe_2_ with broadening diffraction peaks (Fig. 2e). Inasmuch as amorphous MoS_2_ has recently been demonstrated to be effective HER catalysts^13^,^14^,^15^ for its abundant defects and resultant more active edge sites^29^, we thus infer that the quasi-amorphous MoS_2_ modification may benefit the hybrid material to catalytically evolve H_2_. Energy-dispersive X-ray spectrum (EDX; Fig. 2f) analysis further confirmed the formation of MoS_2_/CoSe_2_ hybrid with Co, Se, Mo and S as the principal elemental components (Cu and C peaks emanate from the carbon-coated TEM grid), agreeing with the X-ray photoelectron spectroscopy (XPS) results (Supplementary Fig. 7). Scanning TEM (STEM) and EDX elemental mappings revealed uniform spatial distribution of Co, Se, Mo and S over the marked detection range of the constructed hybrid material (Fig. 2g).

### Catalytic hydrogen evolution

**Figure 3:**
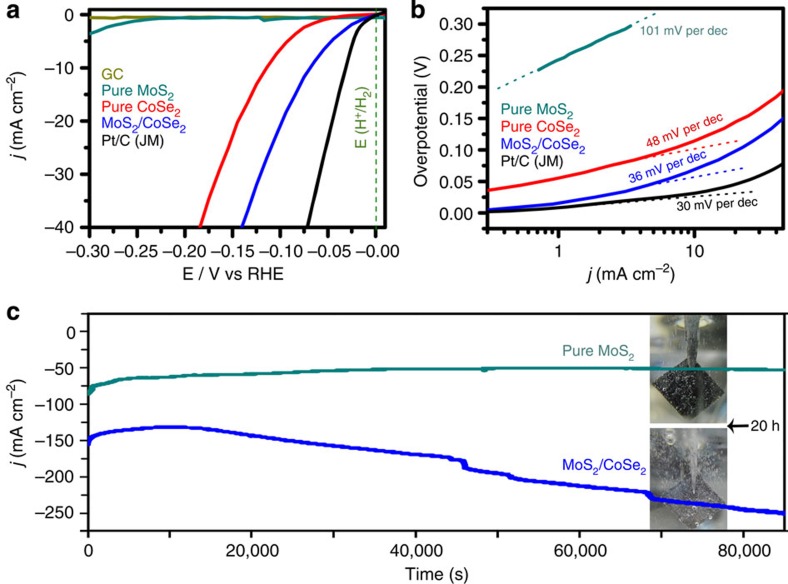
Electrocatalytic hydrogen evolution of different catalysts. (**a**) Polarization curves for HER on bare GC electrode and modified GC electrodes comprising MoS_2_/CoSe_2_ hybrid, pure MoS_2_, pure CoSe_2_ and a high-quality commercial Pt/C catalyst. Catalyst loading is about 0.28 mg cm^−2^ for all samples. Sweep rate: 2 mV s^−1^. (**b**) Tafel plot for the various catalysts derived from **a**. (**c**) Chronoamperometric responses (*j*~*t*) recorded on MoS_2_/CoSe_2_ hybrid and pure MoS_2_ at a constant applied potential of −0.7 V vs SCE. The catalysts were deposited on CFP with the same loading of 1 mg cm^−2^. Inset digital photos show the H_2_ bubbles formed on MoS_2_-modified CFP (up) and MoS_2_/CoSe_2_-modified CFP (down) at the time point of 20 h. All the measurements were performed in H_2_-saturated 0.5 M H_2_SO_4_ electrolyte. dec, decade.

To assess the HER electrocatalytic activity, thin film of various catalysts was prepared on glassy carbon (GC) electrodes for cyclic voltammetry in H_2_-saturated 0.5 M H_2_SO_4_ electrolyte (see Methods for experimental details). Potentials were measured vs saturated calomel electrode (SCE) and are reported vs reversible hydrogen electrode (RHE). The electrode was kept rotating (1,600 r.p.m.) during the measurements to remove *in situ*-emerged H_2_ bubbles. Figure 3a shows that GC electrode guarantees a minimal background activity for H_2_ evolution. A reductive sweep of MoS_2_/CoSe_2_ hybrid showed a low *η* of 11 mV for the HER, beyond which a sharp increase in cathodic current was observed, corresponding to catalytic H_2_ evolution (Fig. 3a; Supplementary Fig. 9). By contrast, pure CoSe_2_ nanobelts exhibited inferior HER activity with a larger onset potential of ~50 mV and lower catalytic current, while pure MoS_2_ nanosheet aggregates only affected little HER activity. The HER kinetics of the above catalysts was probed by corresponding Tafel plots (log *j*~*η*) (Fig. 3b). Tafel slope of ~36 mV per decade was measured for MoS_2_/CoSe_2_ hybrid, which is close to the value of 30 mV per decade for Pt/C catalyst and lower than that of 48 mV per decade for CoSe_2_ and 101 mV per decade for 3D MoS_2_ aggregates (Table 1). This Tafel slope is also comparable to or lower than that of all the reported noble-metal-free HER catalysts in the literature (Supplementary Table 1), demonstrating the superior HER kinetics of MoS_2_/CoSe_2_ hybrid. At high current densities (for example, 10 mA cm^−2^ with *η* of 68 mV), MoS_2_/CoSe_2_ hybrid also represents a more efficient catalyst relative to other noble-metal-free HER catalysts (Supplementary Table 1). The HER inherent activity of these catalysts was evaluated by the exchange current density (*j*_0_). The *j*_0_ of 7.3 × 10^−2^ mA cm^−2^ for MoS_2_/CoSe_2_ hybrid outperforms the values of 8.4 × 10^−3^ mA cm^−2^ for pure CoSe_2_ and 9.1 × 10^−4^ mA cm^−2^ for pure MoS_2_ (Table 1) and also the *j*_0_ value for most of the reported noble-metal-free HER catalysts (Supplementary Table 1). The high electrode kinetic metrics (including onset potential of −11 mV and the Tafel slope of 36 mV per decade) and large *j*_0_ (only ~1 order of magnitude lower than the value of 7.1 × 10^−1^ mA cm^−2^ for Pt) highlight the exceptional H_2_ evolving efficiency of the new MoS_2_/CoSe_2_ hybrid catalyst.

### Material stability

**Figure 4:**
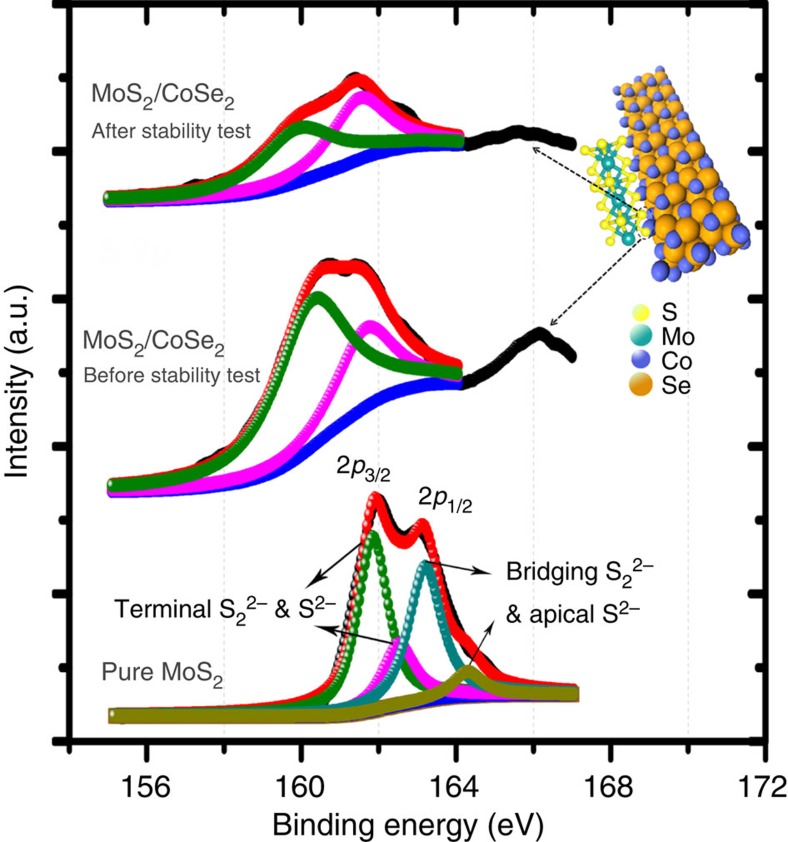
S 2*p* XPS spectrum analysis. S 2*p* XPS spectra for pure MoS_2_, MoS_2_/CoSe_2_ hybrid and MoS_2_/CoSe_2_ hybrid after stability test. The top right corner demonstrates the structure model of MoS_2_/CoSe_2_ hybrid.

The long-term stability of MoS_2_/CoSe_2_ hybrid catalyst was assayed by means of chronoamperometry measurement (*j*~*t*) with a high catalyst loading of 1 mg cm^−2^ on carbon fibre paper (CFP). This quasi-electrolysis process was performed at a constant potential of −0.7 V vs SCE in 0.5 M H_2_SO_4_ for 24 h. As shown in Fig. 3c, the current density of MoS_2_/CoSe_2_-modified CFP electrode decreased gradually at the initial 3 h, which then increased quickly over 24 h of continuous operation. We hypothesize that the more efficient HER-active sites in our hybrid materials are the interfaces between MoS_2_ and CoSe_2_, where Co from the support materials could promote the HER kinetics by lowering the Gibbs free energy of adsorbed hydrogen (Δ*G*_H_)^8^,^29^,^30^. The severe reducing condition at the initial 3 h caused degradation of external MoS_2_ and allowed more electrolyte to access the MoS_2_–CoSe_2_ interfaces (Supplementary Fig. 11; Supplementary Note 1; Supplementary Table 2), yielding the increased HER current density. Remarkably, after 24 h of operation, XPS studies revealed no obvious chemical state change of HER-active S (Fig. 4) and the homogeneous elemental distribution was maintained (Supplementary Fig. 12; Supplementary Note 1), suggesting the robustness of the hybrid catalyst. By comparison, under the exact same condition, the pure MoS_2_ catalyst exhibited a slow but continuous decrease in HER activity. Furthermore, digital photo (inset in Fig. 3c) taken from MoS_2_/CoSe_2_-modified CFP electrode showed vigorous effervescence at 20 h (also Supplementary Movie 1), comparing favourably with the H_2_ bubbles formed on free MoS_2_-modified electrode. The exceptional long-term durability of our MoS_2_/CoSe_2_ hybrid catalyst suggests the promise for implementing this new catalyst into realistic hydrogen evolution electrode.

### HER-enhanced mechanism

The experimentally observed high HER intrinsic activity (*j*_0_=7.3 × 10^−2^ mA cm^−2^) of MoS_2_/CoSe_2_ hybrid catalyst prompted us to probe the enhanced mechanism. Generally, the value of Δ*G*_H_ is considered as a reasonable descriptor of HER activity for a wide variety of catalysts^3^,^30^,^31^. An optimum HER activity is suggested to be achieved at value of Δ*G*_H_≈0 (ref. 30). Lower Δ*G*_H_ will lead to very high surface coverage of *H*_ads_, while higher Δ*G*_H_ will make the protons bonded too weakly on the catalyst surface, which both lead to the slow HER kinetics^3^,^30^,^31^. Previous density functional theory (DFT) calculations^5^ showed that MoS_2_ edge sites with unsaturated sulfur atoms can lower the Δ*G*_H_ approaching to 0 and thus are active for HER, which was later proved by Jaramillo *et al.*^6^ experimentally. Recently, Hu and co-workers^13^,^14^ found that amorphous MoS_2_ films are particularly HER active and the increased coordinately and structurally unsaturated sulfur atoms led to the enhancement. Furthermore, first-row transition metal ions, especially Co, can promote the HER activity of MoS_2_ by coupling with S-edges to lower their Δ*G*_H_ from 0.18 to 0.10 eV to afford a faster proton adsorption kinetics^8^,^29^,^30^. As to MoS_2_/CoSe_2_ hybrid catalyst, the quasi-amorphous MoS_2_ around the CoSe_2_ may bring in more active edge sites. Meanwhile, the CoSe_2_ chemically interacts with MoS_2_ by forming S–Co bond (Fig. 4 and inset), similar to the XPS peak observed in Co-promoted MoS_3_ film^32^, which can further improve the HER activity of MoS_2_. By contrast, no such XPS peak was found in Fig. 4 for pure MoS_2_ catalyst. XPS analyses of the S 2*p* region also exhibit a dramatically decreased electron-binding energy (by ~1.3 eV) after growing MoS_2_ on the CoSe_2_, suggesting the formation of more terminal S_2_^2−^ and S^2−^ ions, which are HER active (Fig. 4)^14^,^19^. Therefore, the CoSe_2_ nanobelts, a material with decent HER activity by itself, can not only chemically couple with MoS_2_ to promote the HER activity, but also serve as an effective support for mediating the growth of MoS_2_ to form more terminal S_2_^2−^ and S^2−^. Meanwhile, anchored MoS_2_ may also boost the HER-active sites of CoSe_2_ and these electrocatalytic synergistic effects^33^ together lead to the high HER performance of MoS_2_/CoSe_2_ hybrid catalyst.

For the HER in acidic media, two separate pathways (the Volmer–Tafel or the Volmer–Heyrovsky mechanism) have been proposed for reducing H^+^ to H_2_ (refs 30, 34). Specifically, the two distinct mechanisms involve three principal steps, referring to the Volmer (electrochemical hydrogen adsorption: H_3_O^+^+e^−^→H_ads_+H_2_O), the Heyrovsky (electrochemical desorption: H_ads_+H_3_O^+^+e^−^→H_2_^↑^+H_2_O) and the Tafel (chemical desorption: H_ads_+H_ads_→H_2_^↑^) reactions^30^,^34^. Tafel slope, an intrinsic property of electrocatalysts, could be used to probe the elementary steps involved in the H_2_ evolution. For example, HER kinetic models suggest that Tafel slope of about 120, 40 or 30 mV per decade will be obtained if the Volmer, Heyrovsky or Tafel reaction is the rate-determining step, respectively^34^. The Tafel slope down to ~36 mV per decade for MoS_2_/CoSe_2_ in 0.5 M H_2_SO_4_ is the lowest value measured till now for MoS_2_-based HER catalysts (Supplementary Table 1), even approaching to that of ~30 mV per decade for Pt/C catalysts. This Tafel slope suggests a Tafel-step-determined Volmer–Tafel mechanism works in the MoS_2_/CoSe_2_ catalyst, where the synergistic CoSe_2_ substrate with decent HER activity presumably contributes to this HER mechanism.

**Figure 5:**
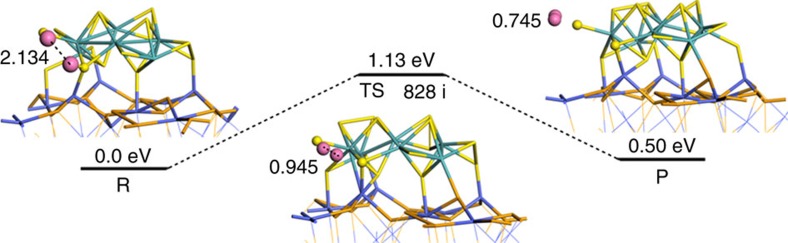
HER mechanism. Reaction pathway of HER on MoS_2_/CoSe_2_ hybrid according to the Volmer–Tafel route. The calculated distance of two hydrogen atoms and energies are displayed in Å and eV. Blue, orange, azure, yellow and pink indicate Co, Se, Mo, S and H atoms, respectively.

Considering that at least one decade of linearity in Tafel extrapolation at relatively large *η* is desirable to ensure an accurate Tafel analysis, the calculated Tafel slopes (Fig. 3b) and derived HER mechanism may be inconclusive. To prove the proposed HER mechanism, we performed a computational study on the new MoS_2_/CoSe_2_ catalyst to gain detailed insights into the adsorption, activation and reaction processes (Fig. 5; see Supplementary Methods for computational details). DFT calculations suggested that reactant (**R**) contains two H atoms adsorbed on different sites of the optimized MoS_2_/CoSe_2_ model with H–H distance of 2.134 Å. Two approached H atoms on the side S atoms of MoS_2_ cluster then formed a transition state (**TS**) with H–H distance of 0.945 Å and imaginary vibration frequency of 828*i* cm^−1^. After crossing the **TS**, product (**P**) with a weakly absorbed H_2_ molecule (0.745 Å) was formed. The calculated activation barrier of 1.13 eV (30.7 kcal mol^−1^) for the Tafel-step reaction on MoS_2_/CoSe_2_ hybrid, which can be overcome at a slightly higher *η*, approaches that of Pt (111) electrode^35^, agreeing well with our experimentally observed fast HER kinetics.

## Discussion

In conclusion, we demonstrate that an effective and robust hydrogen evolution catalyst can be made by marrying inexpensive transition metal chalcogenides. The new MoS_2_/CoSe_2_ catalyst shows exceptional HER catalytic properties in acidic electrolyte with onset potential of mere −11 mV, a small Tafel slope of 36 mV per decade and a high exchange current density, representing the first non-noble metal catalyst that approaches the performance of state-of-the-art Pt/C catalyst. Inspired by the Nature using transition metals as catalytically active site to construct effective HER catalysts (for example, hydrogenase enzymes)^5^,^36^, we believe that our study here will facilitate the development of newly efficient HER catalysts based on transitional metal chalcogenides.

## Methods

### Synthesis of CoSe_2_/DETA nanobelts and MoS_2_/CoSe_2_ hybrid

All chemicals are of analytical grade and were used as received without further purification. First, ultrathin lamellar mesostructured CoSe_2_/DETA nanobelts were prepared by our recently developed method^20^. Briefly, 0.249 g Co(OAc)_2_·H_2_O and 0.173 g Na_2_SeO_3_ were added into a mixed solution (40 ml) with a volume ratio of *V*_DETA_/*V*_DIW_=2:1 (DIW, deionized water). The obtained wine solution was then transferred into a 50 ml Taflon-lined autoclave, which was sealed and maintained at 180 °C for 16 h. The resulting black floccules were collected by centrifugation (4,000 r.p.m. for 5 min) and washed by absolute ethanol for three times, and the resulting CoSe_2_/DETA nanobelts were dried for next use. To prepare the MoS_2_/CoSe_2_ hybrid, 10 mg freshly made CoSe_2_/DETA nanobelts and 10 mg (NH_4_)_2_MoS_4_ were dispersed in 10 ml DMF and sonicated for 15 min under ambient conditions. Then, 0.05 ml N_2_H_4_·H_2_O was added into the suspension. After sonicating for another 15 min to dissolve completely, the mixed solution was transferred into a 50 ml Teflon-lined autoclave, which was sealed and heated in an oven at 200 °C for 10 h and then cooled to room temperature naturally. The resulting black product was collected by centrifugation (7,000 r.p.m. for 8 min), then washed at least four times by distilled water and absolute ethanol to remove ions and possible remnants, and dried under vacuum at 80 °C for 6 h.

### Synthesis of free 3D MoS_2_ sheet aggregates

The synthetic procedure of free 3D MoS_2_ sheet aggregates is the same with that for preparing MoS_2_/CoSe_2_ hybrid, the only difference is that no CoSe_2_/DETA nanobelt substrates were added during the synthesis.

### Characterization

The samples were characterized by different analytic techniques. X-ray powder diffraction (XRD) was carried out on a Rigaku D/max-rA X-ray diffractometer with Cu Ka radiation (*λ*=1.54178 Å); TEM images, HRTEM images, SAED and an energy-disperse X-ray spectrum (EDS) were taken with a JEOJ-2010 transmission electron microscope with an acceleration voltage of 200 kV. STEM and EDX elemental mapping were performed on JEOL ARM-200F. The X-ray photoelectron spectra (XPS) were recorded on an ESCALab MKII XPS using Mg Ka radiation exciting source. The Fourier transform infrared spectra were measured on a Bruker Vector-22 FT-IR spectrometer at room temperature. Nitrogen sorption was determined by Brunauer, Emmett and Teller measurements with an ASAP-2020 surface area analyzer (see Supplementary Fig. 8 for results). The degradation of external MoS_2_ around CoSe_2_ was determined by the inductively coupled plasma–atomic emission spectrometry analysis using an Atomscan Advantage (Thermo Ash Jarrell Corporation, USA) spectrometer.

### Electrocatalytic study

Electrochemical measurements were performed at room temperature using a rotating disk working electrode made of GC (PINE, 5 mm diameter, 0.196 cm^2^) connected to a Multipotentiostat (IM6ex, ZAHNER elektrik, Germany). The GC electrode was polished to a mirror finish (No. 40-6365-006, Gamma Micropolish Alumina, Buehler; No.40-7212, Microcloth, Buehler) and thoroughly cleaned before use. Pt wire and SCE were used as counter and reference electrodes, respectively. The potentials reported in our work were vs the RHE through RHE calibration described below.

The preparation method of the working electrodes containing investigated catalysts can be found as follows. In short, 5 mg of catalyst powder was dispersed in 1 ml of 3:1 v/v DIW/isopropanol mixed solvent with 40 μl of Nafion solution (5 wt%, Sigma-Aldrich), then the mixture was ultrasonicated for about 30 min to generate a homogeneous ink. Next, 10 μl of the dispersion was transferred onto the GC disk, leading to the catalyst loading ~0.28 mg cm^−2^. Finally, the as-prepared catalyst film was dried at room temperature. For comparison, bare GC electrode that has been polished and cleaned was also dried for electrochemical measurement.

Before the electrochemical measurement, the electrolyte (0.5 M H_2_SO_4_) was degassed by bubbling pure hydrogen for at least 30 min to ensure the H_2_O/H_2_ equilibrium at 0 V vs RHE at a rotation rate of 1,600 r.p.m. The polarization curves were obtained by sweeping the potential from −0.7 to −0.2 V vs SCE at room temperature and 1,600 r.p.m. (to remove the *in situ*-formed H_2_ bubbles on the RDE), with a sweep rate of 2 mV s^−1^. The electrochemical impedance spectroscopy measurement was performed in the same configuration at open circuit potential over a frequency range from 100 kHz to 5 mHz at the amplitude of the sinusoidal voltage of 5 mV and room temperature (see Supplementary Fig. 10 for results). MoS_2_/CoSe_2_ and pure MoS_2_-coated CFPs (Toray, 1 cm^2^, catalyst loading 1 mg) were used as working electrodes to collect chronoamperometry data at the applied potential of −0.7 V vs SCE. The polarization curves were replotted as overpotential (*η*) vs log current (log *j*) to get Tafel plots for assessing the HER kinetics of investigated catalysts. By fitting the linear portion of the Tafel plots to the Tafel equation (*η*=*b* log (*j*)+*a*), the Tafel slope (*b*) can be obtained. All data were reported without iR compensation.

### RHE calibration

In all measurements, we used SCE as the reference electrode. It was calibrated with respect to RHE. The calibration was performed in the high-purity hydrogen-saturated electrolyte with a Pt foil as the working electrode. Cyclic voltammetry was run at a scan rate of 1 mV s^−1^, and the average of the two potentials at which the current crossed 0 was taken to be the thermodynamic potential for the hydrogen electrode reaction. In 0.5 M H_2_SO_4_ solution, *E*_RHE_=*E*_SCE_+0.28 V.

### DFT calculations

The computational modelling of the adsorption, activation and reaction processes involved in HER on MoS_2_/CoSe_2_ was performed by periodic DFT with the Vienna *Ab-initio* Simulation Package (VASP). MoS_2_/CoSe_2_ model-building details (Supplementary Figs 13–15), HER mechanism and relevant references are provided in the Supplementary Methods.

## Author contributions

S.-H.Y. and M.-R.G. conceived the idea. M.-R.G., Y.-R.Z. and Y.-F.X. planned and performed the experiments, collected and analysed the data. J.-X.L. and J.L. performed the DFT calculations. J.J. and Q.G. assisted with the experiments and characterizations. M.-R.G. and S.-H.Y. co-wrote the manuscript. All authors discussed the results and commented on the manuscript.

## Additional information

**How to cite this article:** Gao, M.-R. *et al.* An efficient molybdenum disulfide/cobalt diselenide hybrid catalyst for electrochemical hydrogen generation. *Nat. Commun.* 6:5982 doi: 10.1038/ncomms6982 (2015).

## Supplementary Material

Supplementary Information Supplementary Figures 1-15, Supplementary Tables 1-3, Supplementary Notes 1, Supplementary Methods and Supplementary References

Supplementary Movie 1 This movie shows the H2 evolution on MoS2/CoSe2 hybrid modified carbon fiber paper (CFP) electrode in the electrochemical cell at an applied potential of -0.7 V vs. SCE. (electrolyte: 0.5 M H2SO4; MoS2/CoSe2 loading: 1 mg cm-2

## Figures and Tables

**Table 1:** Comparison of catalytic parameters of different HER catalysts.

**Catalyst**	**Onset potention (mV vs RHE)**	**Tafel slope (mV per decade)**	**Exchange current density (*****j***_**0**_**, mA cm**^**−2**^)*
MoS_2_	−237	101	9.1 × 10^−4^
CoSe_2_	−50	48	8.4 × 10^−3^
MoS_2_/CoSe_2_	−11	36	7.3 × 10^−2^
Pt/C†	0	30	7.1 × 10^−1^

HER, H_2_ evolution reaction; RHE, reversible hydrogen electrode.

^*^j_0_ were calculated from Tafel curves using extrapolation method.

^†^Johnson-Matthey, 20 wt% Pt/XC-72.
