# Supplementary material for: An efficient molybdenum disulfide/cobalt diselenide hybrid catalyst for electrochemical hydrogen generation
Source: Nat Commun. 2015 Jan 14;6:5982. doi: 10.1038/ncomms6982 (PMC4309426; doi:10.1038/ncomms6982)
Supplement: Supplementary Information — Supplementary Figures 1-15, Supplementary Tables 1-3, Supplementary Notes 1, Supplementary Methods and Supplementary References [file ncomms6982-s1.pdf]

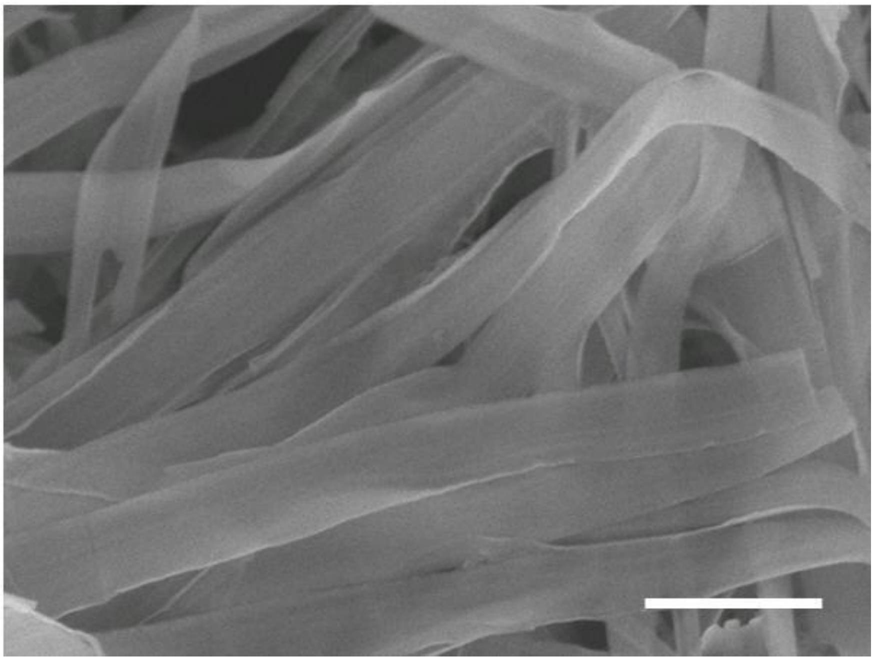

Supplementary Figure 1. SEM characterization. SEM image shows the freshly made $CoSe_2$/DETA nanobelt substrates possess widths of 100-800 nm and lengths up to several tens of micrometers with flexible, smooth, thin and almost transparent features. Scale bar, 2 μm.

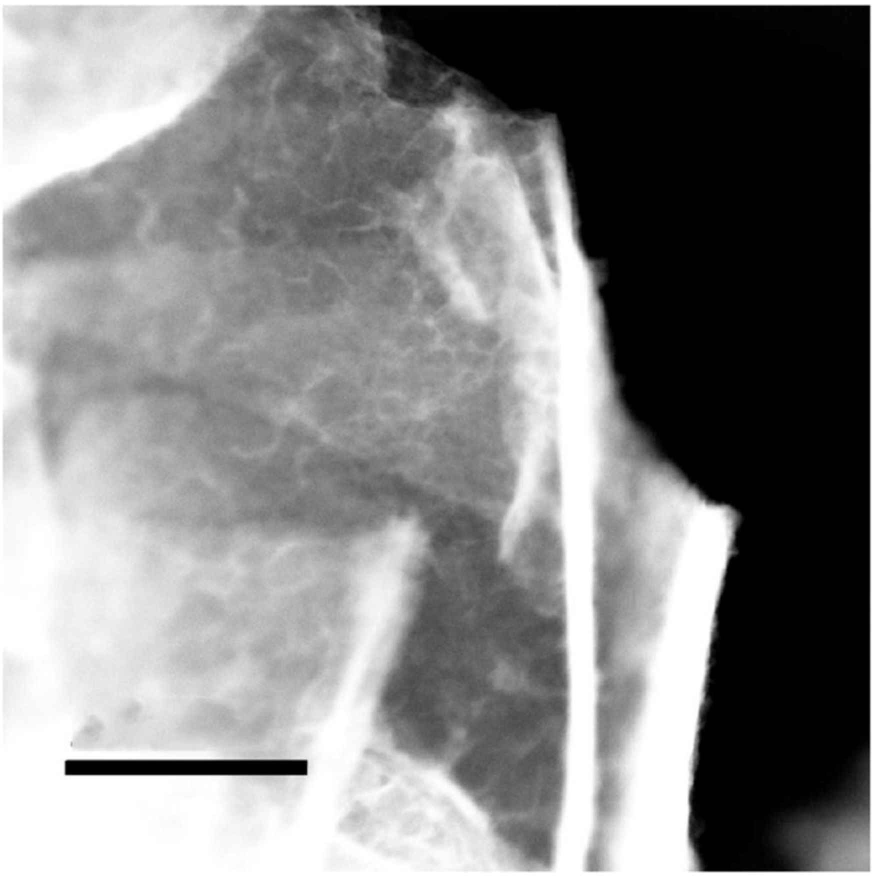

Supplementary Figure 2. STEM characterization. STEM image of $MoS_2/CoSe_2$ hybrid, showing that MoS_2 nanosheets grow around the $CoSe_2$ substrate. Scale bar, 200 nm.

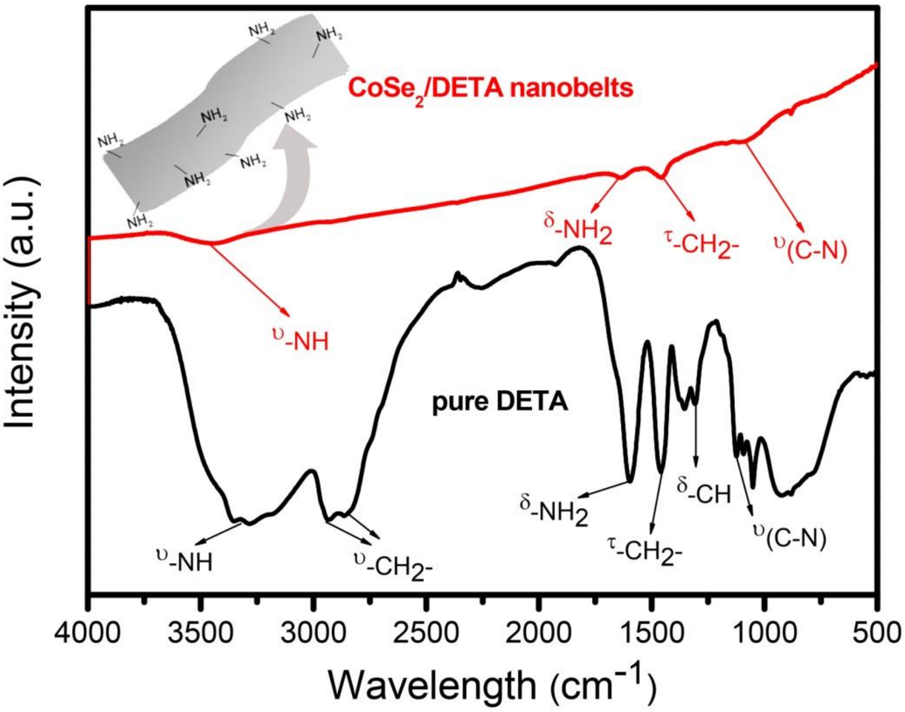

Supplementary Figure 3. Fourier transform infrared (FT-IR) spectra. FT-IR spectra of pure DETA and the $CoSe_2$/DETA nanobelts. The copious amino groups remain on the surface of $CoSe_2$/DETA nanobelts, which can serve as nucleation sites to couple Mo precursors and result in corresponding MoS_2 anchored around the surface of $CoSe_2$ under suitable solvothermal reaction condition.

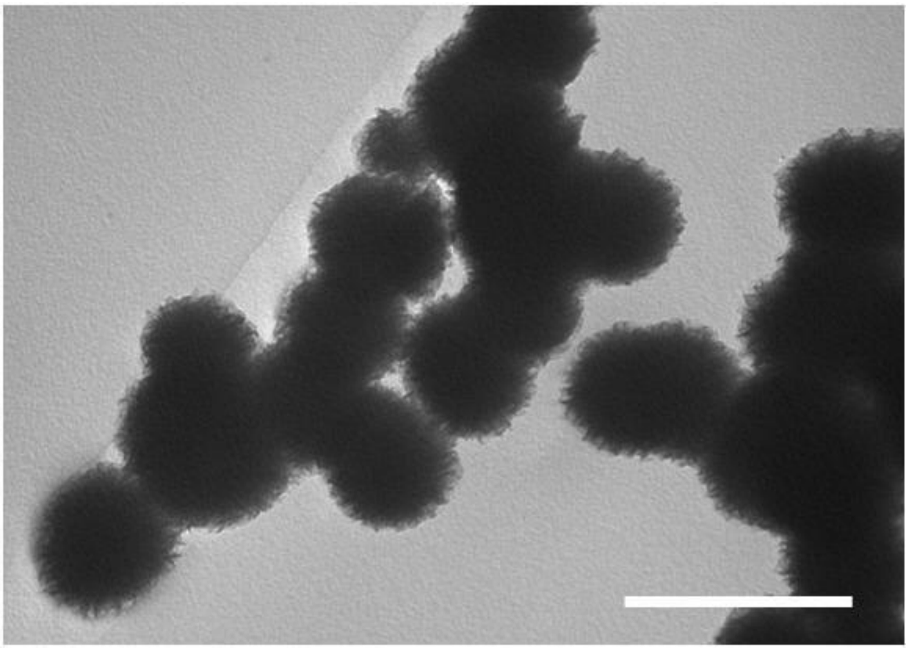

Supplementary Figure 4. TEM characterization. TEM image of free 3D aggregates of MoS_2 sheets without $CoSe_2$ nanobelts as substrates during the solvothermal synthesis. Scale bar, 500 nm.

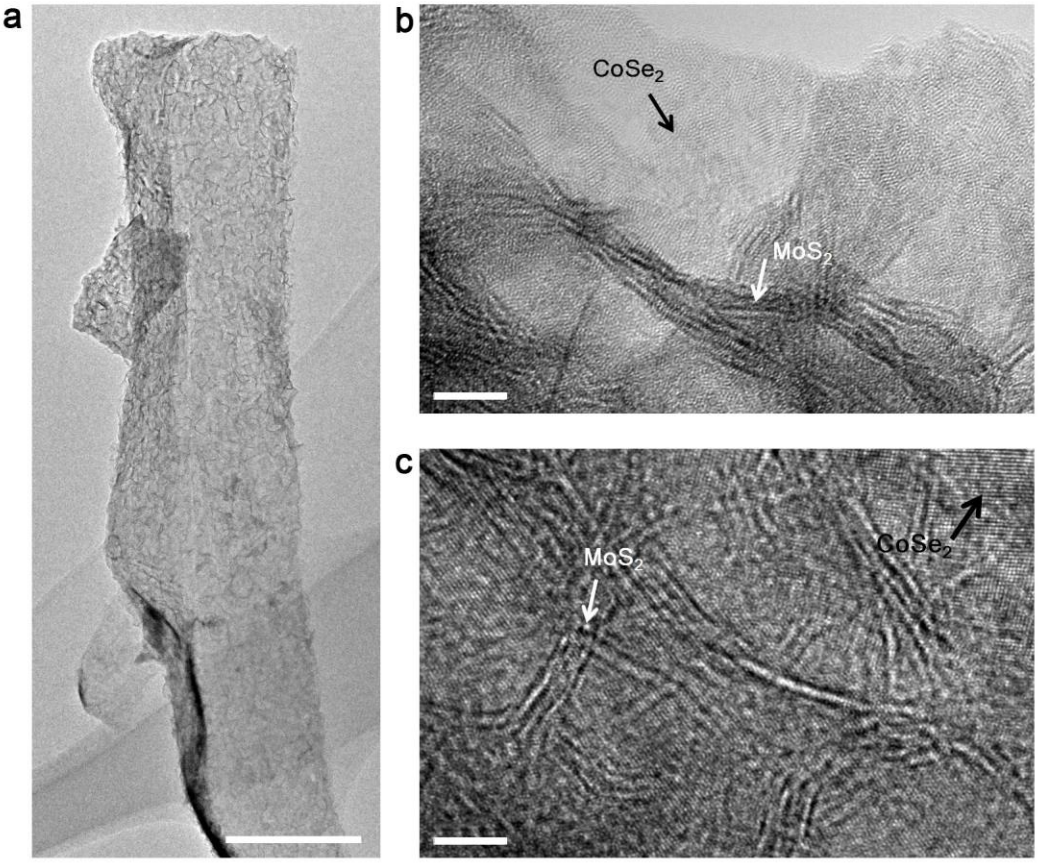

Supplementary Figure 5. TEM and HRTEM images of $MoS_2/CoSe_2$ hybrid. (**a**) TEM image of a typical $MoS_2/CoSe_2$ hybrid nanobelt. Scale bar, 200 nm. (**b,c**) HRTEM images of $MoS_2/CoSe_2$ hybrid, which further reveal the hybrid structure where graphene-like MoS_2 nanosheets are anchored intimately on the surface of $CoSe_2$ substrate. Scale bars, 5 nm.

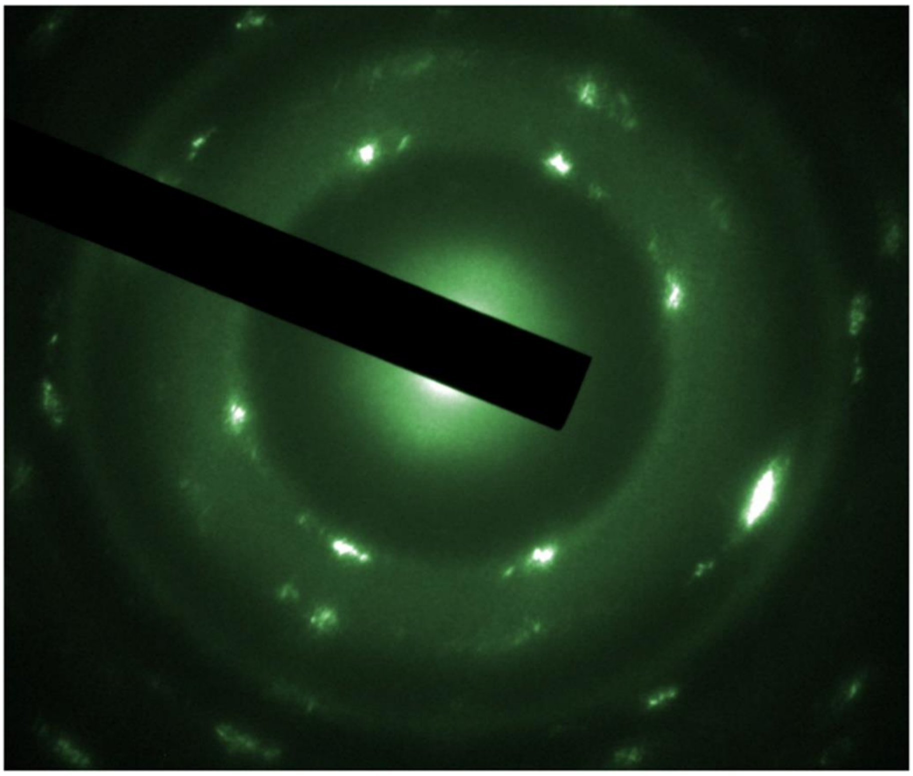

Supplementary Figure 6. SAED pattern. Enlarged SAED pattern taken on a typical $MoS_2/CoSe_2$ hybrid, corresponding to the TEM image and SAED pattern in Fig. 2c.

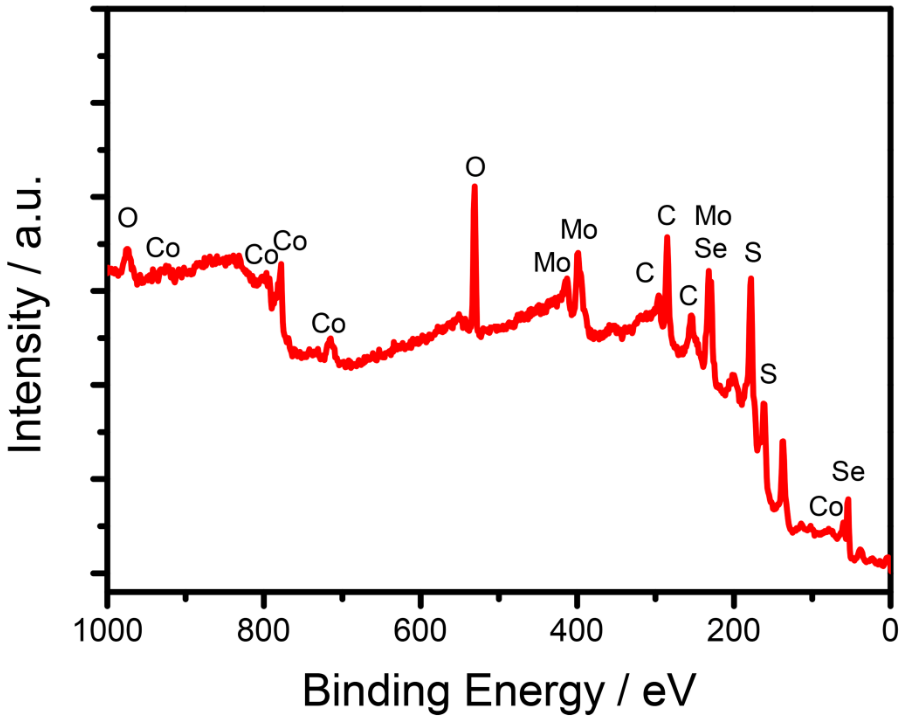

Supplementary Figure 7. XPS characterization. XPS survey spectrum of $MoS_2/CoSe_2$ hybrid.

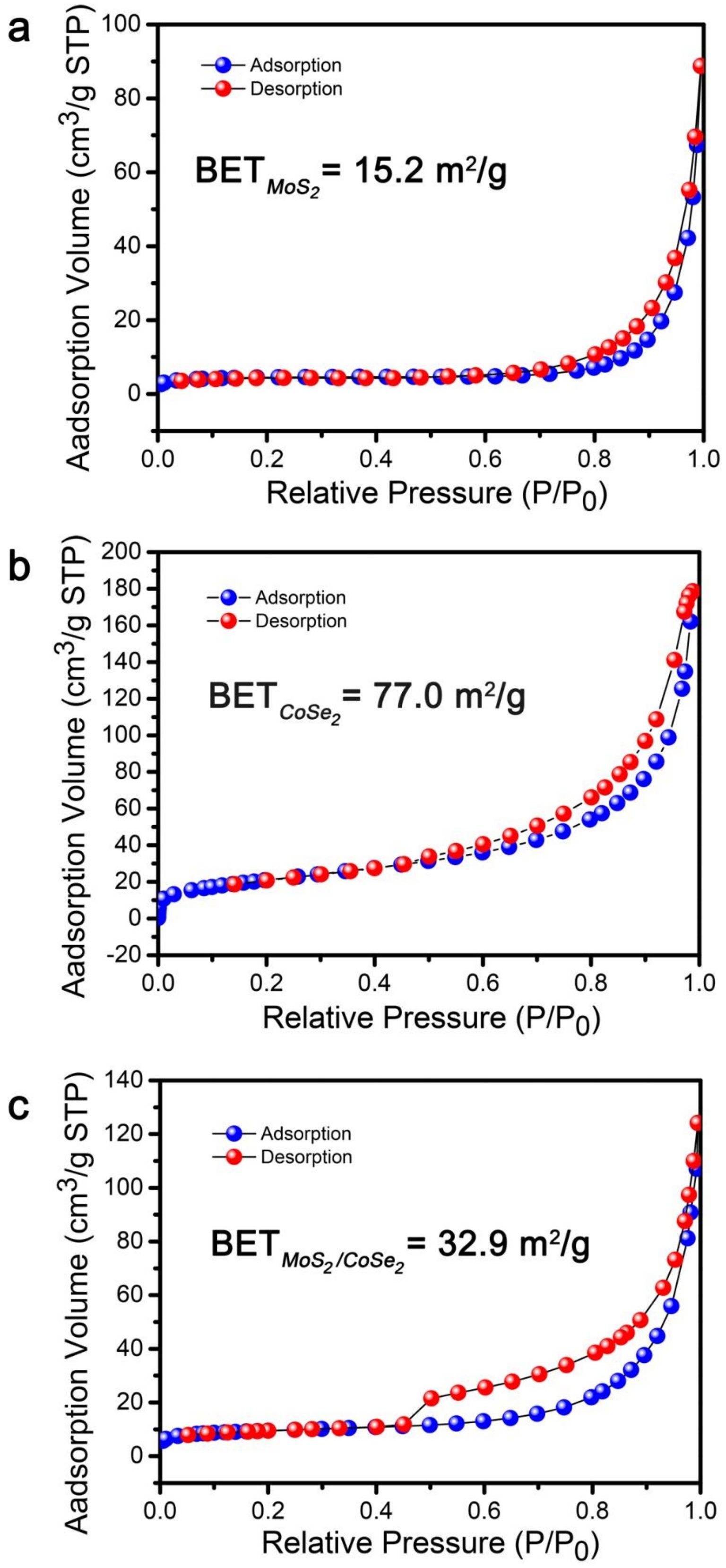

Supplementary Figure 8. BET characterization. (**a,b,c**) Nitrogen adsorption-desorption isotherms for pure 3D MoS_2 nanosheet aggregates, pure $CoSe_2$ nanobelts, and $MoS_2/CoSe_2$ hybrid, respectively. The smaller BET surface area of

$MoS_2/CoSe_2$ hybrid as compared to $CoSe_2$ nanobelts indicated some stacks existed in the $MoS_2/CoSe_2$ sample.

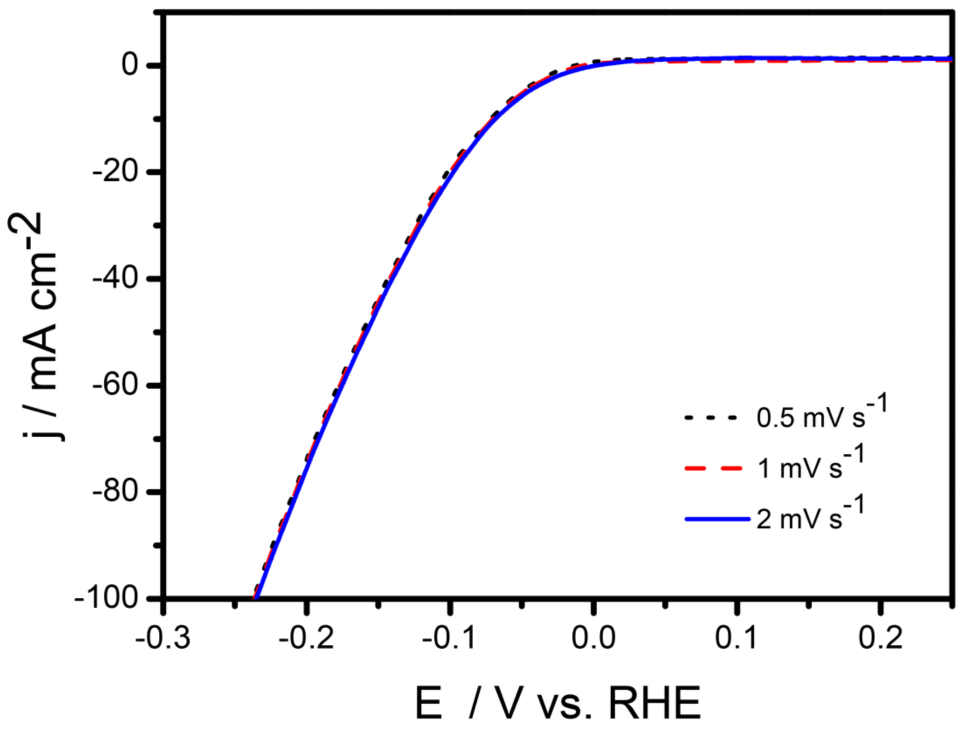

Supplementary Figure 9. HER polarization curves for MoS_2/$CoSe_2$ hybrid catalyst at different sweep rates. It can be seen that the HER polarization curves are almost independent on different slow sweep rates used here, indicating that the sweep rate of 2 mV s^{-1} is slow enough to build a steady state electrode and thus the resulting polarization curve is reasonably to be used for kinetic analysis.

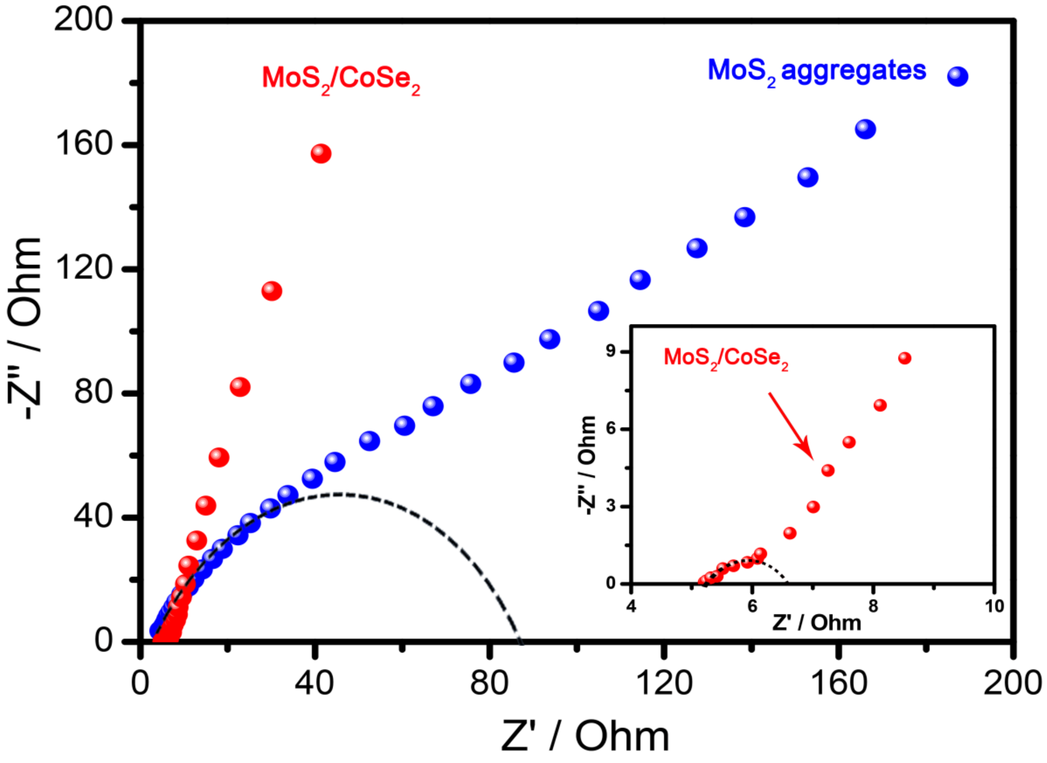

Supplementary Figure 10. EIS Nyquist plots. Nyquist plots of pure 3D MoS_2 and $MoS_2/CoSe_2$ hybrid. Inset shows Nyquist plot at high-frequency range for $MoS_2/CoSe_2$ hybrid. Z' is the real impendence and -Z" is the imaginary impedance. The kinetics of electrode reactions for pure 3D MoS_2 and $MoS_2/CoSe_2$ hybrid were also probed by electrochemical impedance spectroscopy (EIS) technique. The Nyquist plots (Z_{real} vs. $-Z_{im}$) of the two catalysts both consist of a depressed semicircle in the high-frequency region (corresponding to charge transfer resistance, R_{ct}) and a quasi-sloping line in the low-frequency region (corresponding to mass transfer resistance). The obviously much smaller R_{ct} (diameter of the semicircle) value of $MoS_2/CoSe_2$ hybrid electrode suggests its higher charge transport efficiency and thus faster HER kinetics.

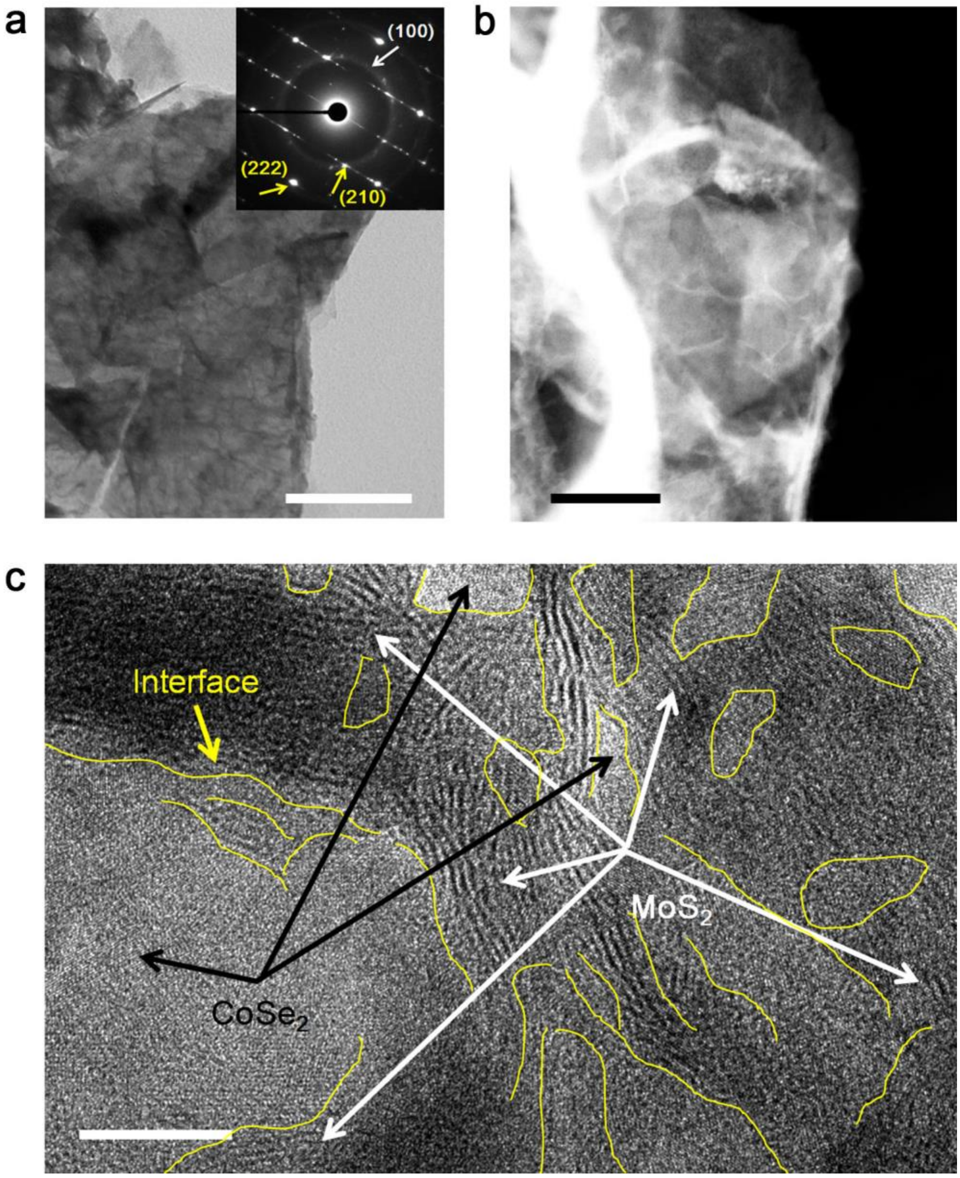

Supplementary Figure 11. TEM, STEM and HRTEM images of $MoS_2/CoSe_2$ hybrid after stability test. (**a,b**) TEM (Scale bar, 200 nm) and STEM (Scale bar, 100 nm) images taken after stability test for $MoS_2/CoSe_2$ hybrid, respectively. The inset in (**a**) shows corresponding SAED pattern. (**c**) HRTEM images of $MoS_2/CoSe_2$ after stability test, where more MoS_2-$CoSe_2$ interfaces were exposed. Scale bar, 10 nm.

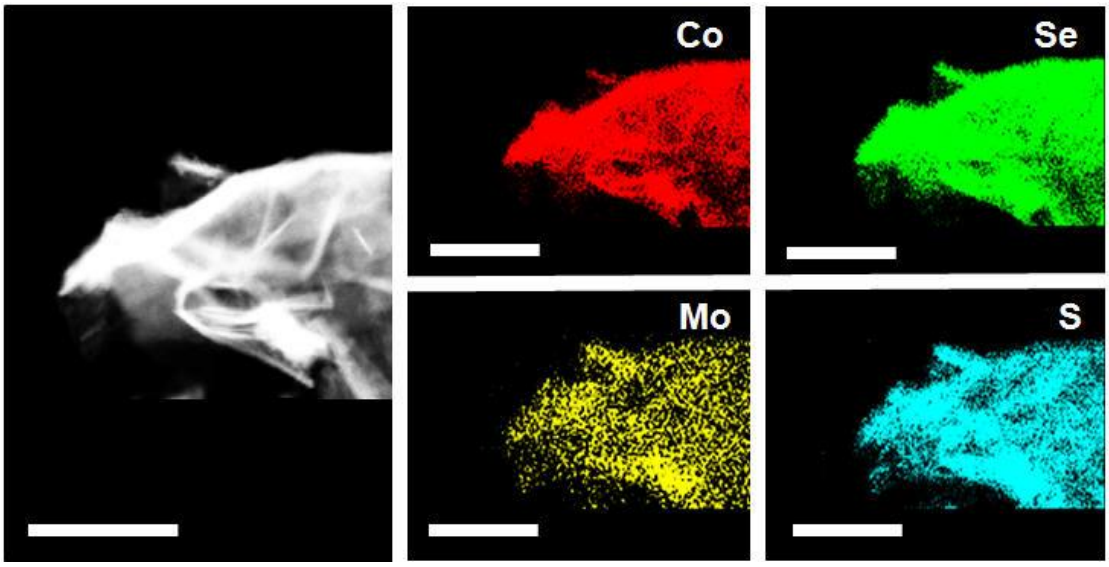

Supplementary Figure 12. STEM-EDX elemental mapping results for $MoS_2/CoSe_2$ hybrid after stability test, suggesting that Co (red), Se (green), Mo (yellow) and S (azure) are maintained with homogeneous distribution. Scale bars, 300 nm.

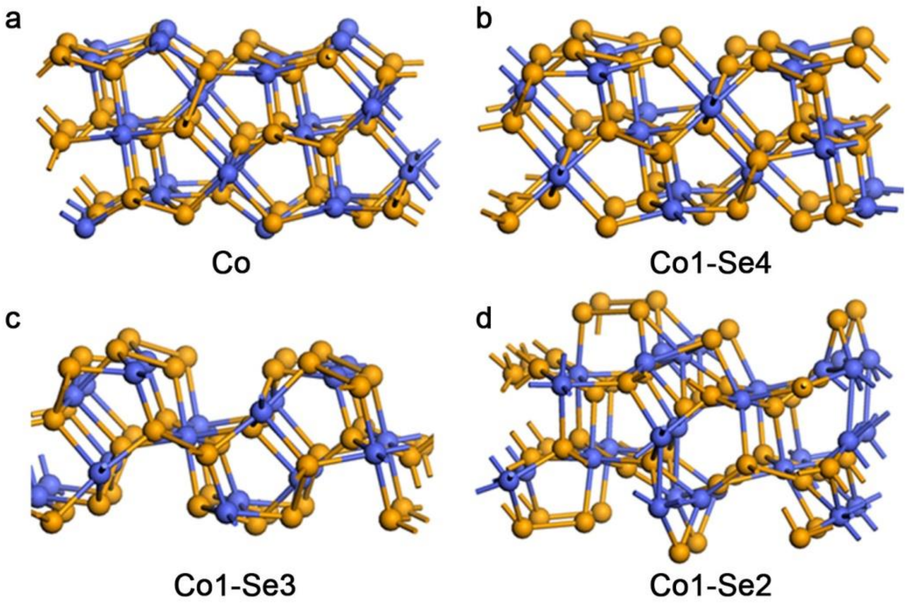

Supplementary Figure 13. Optimized $CoSe_2$ (210) surface with different terminations (**a**) surface-1: Co, (**b**) surface-2: Co1-Se4, (**c**) surface-3: Co1-Se3, and (**d**) surface-4: Co1-Se2. Blue and orange spheres indicate Co and Se atoms, respectively.

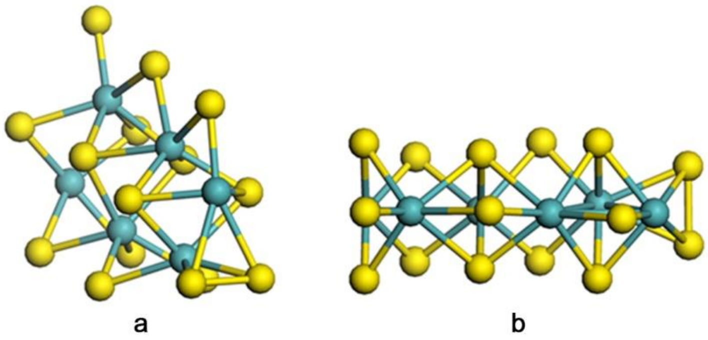

Supplementary Figure 14. Optimized MoS_2 cluster (**a**) one side view, and (**b**) another side view, respectively. Azure and yellow spheres indicate Mo and S atoms, respectively.

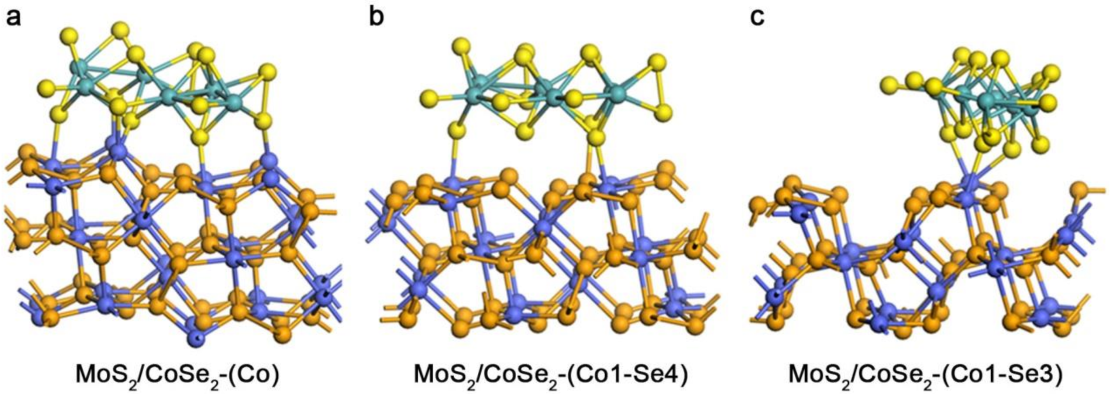

Supplementary Figure 15. Optimized structures of $MoS_2/CoSe_2$ hybrid catalyst with different terminations of the $CoSe_2$ (210) surface. (**a**) on surface-1: $MoS_2/CoSe_2$-(Co), (**b**) on surface-2: $MoS_2/CoSe_2$-(Co1-Se4), and (**c**) on surface-3: $MoS_2/CoSe_2$-(Co1-Se3). Blue, orange, azure, and yellow spheres indicate Co, Se, Mo and S atoms, respectively.

Supplementary Table 1. Summary of literature catalytic parameters of various noble-metal-free HER catalysts.

Catalyst	Electrolyte	Onset potential (mV *vs.* RHE)	Tafel slope (mV dec^{-1})	j_0 (mV cm^{-2})	η @ j = 10 mA cm^{-2} (mV)	Loading	Ref.
Ni_2P Nanoparticles	0.5M H_2SO_4	N/A	46 (η=25~125 mV)	3.3 x 10^{-2}	N/A	1 mg cm^{-2}	1
			81 (η=150~200 mV)	4.9 x 10^{-1}			
FeP porous sheets	0.5M H_2SO_4	-100	67	N/A	N/A	0.28 mg cm^{-2}	2
$NiMoN_x$/C	0.1M $HClO_4$	-78	35.9	2.4 x 10^{-1}	N/A	N/A	3
Mo_2C	1M H_2SO_4	*ca.* -151	56	1.3 x 10^{-3}	*ca.* 214	1.4 mg cm^{-2}	4
MoB	1M H_2SO_4	*ca.* -155	55	1.4 x 10^{-3}	*ca.* 212	2.5 mg cm^{-2}	4
$[Mo_3S_4]^{4+}$	0.5M H_2SO_4	N/A	120	2.2 x 10^{-3}	N/A	0.0067 μg cm^{-2}	5
MoS_2 ultrathin sheets	0.5M H_2SO_4	*ca.* -155	50	8.91 x 10^{-3}	195	0.285 mg cm^{-2}	6
Metallic 1T MoS_2 sheets	0.5M H_2SO_4	*ca.* -135	43	N/A	*ca.*187	N/A	7
Nanoparticulate MoS_2	0.5M H_2SO_4	N/A	55~60	1.3~3.1 x 10^{-3}	N/A	N/A	8
Porous MoS_2 film	0.5M H_2SO_4	N/A	41~45	2.5 x 10^{-4}	N/A	N/A	9
Amorphous MoS_2	1M H_2SO_4	N/A	40	1.3 x 10^{-3}	N/A	N/A	10
Vertically aligned MoS_2 film	0.5M H_2SO_4	*ca.* -200	40	2.2 x 10^{-3}	N/A	8.5 μg cm^{-2}	11
Vertically aligned $MoSe_2$ film			75	2.0 x 10^{-3}		13.5 μg cm^{-2}	
CoS_2 film	0.5M H_2SO_4	N/A	44.6	5.4 x 10^{-5}	N/A	N/A	12
$CoSe_2$ film			42.4	7.5 x 10^{-5}			
NiS_2 film			41.6	1.4 x 10^{-5}			
$NiSe_2$ film			56.9	7.5 x 10^{-4}			
Core/shell C/$CoSe_2$	0.5M H_2SO_4	N/A	40	3.7 x 10^{-2}	N/A	37 μg cm^{-2}	12
NiSe fiber assemblies	0.5M H_2SO_4	-200	64	N/A	230	0.28 mg cm^{-2}	13
Lamellar $CoSe_2$ nanobelts	0.5M H_2SO_4	-50	48	8.4 x 10^{-3}	115	0.28 mg cm^{-2}	14
Ni/ NiO/$CoSe_2$	0.5M H_2SO_4	-30	39	1.4 x 10^{-2}	88	0.28 mg cm^{-2}	14
MoS_x/Ni foam	0.5M H_2SO_4	-130	42.8	N/A	N/A	N/A	15
MoS_2/ graphene	0.5M H_2SO_4	*ca.* -100	41	N/A	*ca.*150	0.28 mg cm^{-2}	16
Pt/MoS_2	0.5M H_2SO_4	N/A	40	N/A	N/A	0.075 mg cm^{-2}	17
MoS_2/Au	0.5M H_2SO_4	-90	69	9.3 x 10^{-3}	N/A	1.03 μg cm^{-2}	18
MoO_3/MoS_2 nanowires	0.5M H_2SO_4	-150~-200	50~60	N/A	*ca.* 240	N/A	19
Mo_2C/carbon nanotubes	0.1M $HClO_4$	N/A	55.2	1.4 x 10^{-2}	152	2 mg cm^{-2}	20
$MoSe_2$/carbon paper	0.5M H_2SO_4	N/A	59.8	3.8 x 10^{-4}	250	N/A	21
WSe_2/carbon paper			77.4	N/A	300		
MoS_2/WC/RGO	0.5M H_2SO_4	-110	41	N/A	N/A	0.104 mg cm^{-2}	22
MoS_2/graphene foam	0.5M H_2SO_4	-150	42	N/A	N/A	0.21 mg cm^{-2}	23
Co-promoted MoS_2 film	1M H_2SO_4	N/A	43	5.0 x 10^{-4}	N/A	N/A	24
MoS_2/$CoSe_2$ hybrid	0.5M H_2SO_4	-11	36	7.3 x 10^{-2}	68	0.28 mg cm^{-2}	This work

Supplementary Table 2. ICP results show the concentration of dissolved elements in electrolyte after stability test.

**Element**	**Co**	**Se**	**Mo**	**S**
**Amount (μg/ml)**	0.289	0.862	64.200	52.387

Supplementary Table 3. Binding energies BE (in eV) for MoS_2 cluster and the average bond lengths for S-Co (in Å) of MoS_2 cluster adsorbed on the Co, Co-Se, and Co1-Se3 surfaces.*

**Surface**	**BE**	**Average Bond length (S-Co/Å)**
**Co**	4.23	2.227
**Co1-Se4**	0.24	2.320
**Co1-Se3**	3.08	2.338

*The binding energy is calculated as $BE_{MoS2/CoSe2} = E[MoS_2]+E[CoSe_2]-E[MoS_2/CoSe_2]$

Supplementary Note 1

Structural and chemical analyses performed after a 24-h electrolysis experiment on $MoS_2/CoSe_2$-modified-CFP electrode gain useful insights into the extreme robustness of the hybrid material. **Supplementary Figure 11a,b** (with inset SAED pattern) reveal that MoS_2-coated $CoSe_2$ hybrid structure was maintained after 24-h testing, whereas more MoS_2-$CoSe_2$ interfaces were exposed (Supplementary Fig. 11c) due to the partial corrosion of MoS_2 from the hybrid surface (**Supplementary Table. 2**). This implies that the Co-promoted interfaces are more efficient active sites for reducing water. Although the as-tested sample exhibited an increase in surface roughness (Supplementary Figure 11a,b), our STEM-EDX results demonstrated a homogeneous elemental distribution even after 24 hours of operation (**Supplementary Figure 12**). Moreover, comparing with the freshly prepared $MoS_2/CoSe_2$ sample, we did not detect obvious chemical state change of HER active S in as-tested sample by X-ray photoelectron spectroscopy (XPS), further supporting the remarkable stability of this hybrid material (**Figure 4 in the main text**).

Supplementary Methods

The computational modeling of the adsorption, activation and reaction processes involved in HER on the new catalyst was performed by periodic density functional theory (DFT) with the Vienna Ab-initio Simulation Package (VASP) [25,26].

From the experimental results, we found that MoS_2 nanosheets only partially covered around the single-crystalline $CoSe_2$ support. We therefore designated a model with selected MoS_2 clusters anchored onto 2D periodic slab of $CoSe_2$ nanostructure. The optimized bulk cell of $CoSe_2$ has a = b = c = 5.860 Å, which is close to the experimental data of 5.854 Å[27]. 2D slab model of different termination surfaces of $CoSe_2$ nanostructure was obtained by appropriately cutting the stable pyrite structure with $CoSe_2$ (210) surface, which led to a rectangular unit cell of 13-17 atomic layers (11.7×13.1 Å^2, more than 60 atoms). The bottom 4-7 atomic layers were frozen and the other top-layer slabs of the surface were allowed to relax during the geometry optimizations. The periodically repeated slabs were separated from their neighboring images by a 12 Å-width vacuum in the direction perpendicular to the surface. The selection of the $CoSe_2$ surfaces and MoS_2/$CeSe_2$ model are described later together with the results (see below).

The core and valence electrons of Mo, Co, Se, and S atoms were represented by the projector augmented wave (PAW) method[28] and plane-wave basis functions with a kinetic energy cut-off of 280 eV. The generalized gradient approximation (GGA) with the Perdew-Burke-Ernzerhof (PBE)[29] exchange-correlation functional was used in all the calculations. A Monkhorst-Pack grid of size of 2×2×1 was used to sample

the surface Brillouin zone. Ground-state atomic geometries were obtained by minimizing the forces on the atoms to below 0.02 eV/Å. The transition states were obtained by relaxing the force below 0.02 eV/Å by using the dimer method[30].

The stability of $CoSe_2$ (210) surface with different terminations. $CoSe_2$ (210) surface has six different terminations like pyrite FeS_2[31], here we considered four different terminations containing Co on the surfaces and the optimized structures are displayed in **Supplementary Figure 13**. As seen from Supplementary Figure 13, surface-1 only contains 3-fold Co on the outermost layer (Co), surface-2 contains Co surrounding by four Se atoms (Co1-Se4), surface-3 contains Co surrounding by three Se atoms (Co1-Se3), and surface-4 contains Co surrounding by two Se atoms (Co1-Se2). Among these four structures, significant surface reconstruction was found for Co1-Se2, indicating its low stability. Accordingly, we only investigated the binding energies of MoS_2 cluster anchored on the Co, Co1-Se4, and Co1-Se3 surfaces in the subsequent calculations.

The structure model of hybrid catalyst $MoS_2/CoSe_2$. Based on previous calculations[32], we constructed a MoS_2 cluster and its optimized structure was shown in **Supplementary Figure 14**. The stable MoS_2 cluster was anchored on the three aforementioned surfaces through the bonding between S from MoS_2 and Co from these $CoSe_2$ surfaces. The optimized hybrid structures are shown in **Supplementary Figure 15** and the binding energies (BE, in eV) for MoS_2 cluster, the average bond

lengths for S-Co (in Å) of MoS_2 cluster adsorbed on the Co, Co-Se, and Co1-Se3 surfaces were collected in **Supplementary Table 3**. As seen from Supplementary Table 3, the average bond length of S-Co in $MoS_2/CoSe_2$-(Co) is 2.227 Å, which was shorter than those in $MoS_2/CoSe_2$-(Co1-Se4) (2.320 Å) and $MoS_2/CoSe_2$-(Co1-Se3) (2.338 Å). These data indicated that the MoS_2 cluster has stronger interaction with the surface-1 (Co). Indeed, the binding energy of MoS_2 cluster on the surface Co was calculated to be 4.23 eV, which was significantly larger than those involving other surfaces.

Supplementary references

1 Popczun, E. J. et al. Nanostructured nickel phosphide as an electrocatalyst for the hydrogen evolution reaction. *J. Am. Chem. Soc.* **135,** 9267-9270 (2013).

2 Xu, Y., Wu, R., Zhang, J. F., Shi, Y. M., & Zhang, B. Anion-exchange synthesis of nanoporous FeP nanosheets as electrocatalysts for hydrogen evolution reaction. *Chem. Commun.* **49,** 6656-6658 (2013).

3 Chen, W. F. *et al.* Hydrogen-evolution catalysts based on non-noble metal nickel-molybdenum nitride nanosheets. *Angew. Chem. Int. Ed.* **51,** 6131-6135 (2012).

4 Vrubel, H. & Hu, X. L. Molybdenum boride and carbide catalyze hydrogen evolution in both acidic and basic solutions. *Angew. Chem. Int. Ed.* **51,** 12875-12878 (2012).

5 Jaramillo, T. F. *et al.* Hydrogen evolution on supported incomplete cubane-type $[Mo_3S_4]^{4+}$ electrocatalysts. *J. Phys. Chem. C* **112,** 17492-17498 (2008).

6 Xie, J. F. *et al.* Defect-rich MoS_2 ultrathin nanosheets with additional active edge sites for enhanced electrocatalytic hydrogen evolution. *Adv. Mater.* **25,** 5807-5813 (2013).

7 Lukowski, M. A. *et al.* Enhanced hydrogen evolution catalysis from chemically exfoliated metallic MoS_2 nanosheets. *J. Am. Chem. Soc.* **135,** 10274-10277 (2013).

8 Jaramillo, T. F. *et al.* Identification of active edge sites for electrochemical H_2 evolution from MoS_2 nanocatalysts. *Science* **317,** 100-102 (2007).

9 Lu, Z. Y. *et al.* In situ fabrication of porous MoS_2 thin-films as high-peformance catalysts for electrochemical hydrogen evolution. *Chem. Comm.* **49,** 7516-7518 (2013).

10 Merki, D., Fierro, S., Vrubel, H. & Hu, X. L. Amorphous molybdenum sulfide films as catalysts for electrochemical hydrogen production in water. *Chem. Sci.* **2,** 1262-1267 (2011).

11 Kong, D. S. *et al.* Synthesis of MoS_2 and $MoSe_2$ films with vertically aligned layers. *Nano. Lett.* **13,** 1341-1347 (2013).

12 Kong, D. S., Cha, J. J., Wang, H. T., Lee, H. R. & Cui, Y. First-row transition metal dichalcogenide catalysts for hydrogen evolution reaction. *Energy Environ. Sci.* **7,** 3553-3558 (2013).

13 Gao, M. R. *et al.* Mixed-solution synthesis of sea urchin-like NiSe nanofiber assemblies as economical Pt-free catalysts for electrochemical H_2 production. *J. Mater. Chem.* **22,** 13662-13668 (2012).

14 Xu, Y. F., Gao, M. R. Zheng, Y. R., Jiang, J. & Yu, S. H. Nickel/nickel(II) oxide nanoparticles anchored onto cobalt(IV) diselenide nanobelts for the electrochemical production of hydrogen. *Angew. Chem. Int. Ed.* **52,** 8546-8550 (2013).

15 Chang, Y. H. *et al.* Highly efficient electrocatalytic hydrogen production by MoS_x grown on graphene-protected 3D Ni forams. *Adv. Mater.* **25,** 756-760 (2013).

16 Li, Y. G. *et al.* MoS_2 nanoparticles grown on graphene: an advanced catalyst for the hydrogen evolution reaction. *J. Am. Chem. Soc.* **133,** 7296-7299 (2011).

17 Huang, X. *et al.* Solution-phase epitaxial growth of noble metal nanostructures on dispersible single-layer molybdenum disulfide nanosheets. *Nat. Commun.* **4,** DOI: 10.1038/ncomms2472 (2013).

18 Wang, T. Y. *et al.* Enhanced electrocatalytic activity for hydrogen evolution reaction from self-assembled monodispersed molybdenum sulfide nanoparticles on an Au electrode. *Energy Environ. Sci.* **6,** 625-633 (2013).

19 Chen, Z. B. *et al.* Core-Shell MoO_3-MoS_2 nanowires for hydrogen evolution: a functional design for electrocatalytic materials. *Nano Lett.* **11,** 4168-4175 (2011).

20 Chen, W. F. *et al.* Highly active and durable nanostructured molybdenum carbide electrocatalysts for hydrogen production. *Energy Environ. Sci.* **6,** 943-951 (2013).

21 Wang, H. T. *et al.* $MoSe_2$ and WSe_2 nanofilms with vertically aligned molecular layers on curved and rough surfaces. *Nano Lett.* **13,** 3426-3433 (2013).

22 Yan, Y. *et al.* Nano-tungsten carbide decorated graphene as co-catalysts for enhanced hydrogen evolution on molybdenum disulfide. *Chem. Commun.* **49,** 4884-4886 (2013).

23 Liao, L. *et al.* MoS_2 formed on mesoporous graphene as a highly active catalyst for hydrogen evolution. *Adv. Funct. Mater.* **23,** 5326-5333 (2013).

24 Merki, D., Vrubel, H., Rovelli, L., Fierro, S. & Hu, X. L. Fe, Co, and Ni ions promote the catalytic activity of amorphous molybdenum sulfide films for hydrogen evolution. *Chem. Sci.* **3,** 2515-2525 (2012).

25 Kresse, G., & Hafner, J. Ab initio molecular dynamics for liquid metals *Phys. Rev. B*, **47**, 558-561 (1993).

26 Kresse, G., & Joubert, D. From ultrasoft pseudopotentials to the projector augmented-wave method. *Phys. Rev. B*, **59**, 1758-1775 (1999).

27 Mande, C., & Nigavekar, A. S. X-ray spectroscopic study of chemical bonding in $MnSe_2$ and $CoSe_2$ [C]//Proceedings of the Indian Academy of Sciences-Section A. Springer India, **67**(3): 166-174 (1968).

28 Blöchl, P. E. Projector augmented-wave method. *Phys. Rev. B*, **50**, 17953-17979 (1994).

29 Perdew, J. P., Burke, K. & Ernzerhof, M.. Generalized gradient approximation made simple. *Phys. Rev. Lett.*,**77,** 3865-3868 (1996).

30 Henkelman, G., & Jónsson, H. A dimer method for finding saddle points on high dimensional potential surfaces using only first derivatives. *J. Chem. Phys.*, **111**, 7010-7022 (1999).

31 Alfonso, D. R. Computational investigation of FeS_2 surfaces and prediction of effects of sulfur environment on stabilities. *J. Phys. Chem. C* **114**, 8971-8980 (2010).

32 Jean-Francüois, P. & Edmond, P. Vacancy formation on MoS_2 hydrodesulfurization catalyst: DFT study of the mechanism. *J. Phys. Chem. B* **107**, 4057-4064 (2003).
